# Novel mucosal adjuvant, mastoparan-7, improves cocaine vaccine efficacy

**DOI:** 10.1038/s41541-020-0161-1

**Published:** 2020-02-05

**Authors:** Ashley L. St. John, Hae Woong Choi, Q. David Walker, Bruce Blough, Cynthia M. Kuhn, Soman N. Abraham, Herman F. Staats

**Affiliations:** 1 Program in Emerging Infectious Diseases, Duke-National University of Singapore, Singapore, 169857 Singapore; 2 Department of Pathology, Duke University Medical Center, Durham, NC 27710 USA; 3 Department of Microbiology and Immunology, Yong Loo Lin School of Medicine, National University of Singapore, Singapore, 119228 Singapore; 4 SingHealth Duke-NUS Global Health Institute, Singapore, 168753 Singapore; 5 Department of Pharmacology and Cancer Biology, Duke University Medical Center, Durham, NC 27710 USA; 6 Center for Drug Discovery, RTI International, Research Triangle Park, Durham, NC 27709 USA; 7 Department of Immunology, Duke University Medical Center, Durham, NC 27710 USA; 8 Department of Molecular Genetics and Microbiology, Duke University Medical Center, Durham, NC 27710 USA; 9 Duke Human Vaccine Institute, Duke University Medical Center, Durham, NC 27710 USA; 10 Present Address: Korea University, Division of Life Sciences, 108 Hana-Science Building, 145 Anam-ro, Seongbuk-gu, Seoul, South Korea

**Keywords:** Conjugate vaccines, Addiction, Vaccines

## Abstract

Cocaine is one of the most potent and addictive psychostimulants known and there are no available pharmacotherapies to treat cocaine addiction. Here we describe a novel cocaine vaccine employing the mucosal adjuvant and mast cell-activating oligopeptide, mastoparan-7 (M7), to achieve optimal IgA antibody responses in mucosal secretions and effective induction of humoral immunity using a short immunization protocol. This formulation, using a hapten-carrier system to deliver cocaine as antigen, also reduced cocaine penetration of the blood brain barrier and protected mice from its psychoactive effects by reducing cocaine-induced locomotion. Surprisingly, the magnitude of cocaine-specific antibody titers induced by each adjuvant was not the major determinant of functional protection from cocaine challenge. A side-by-side comparison of the two haptens, cocaine and its analog GNC demonstrated that cocaine haptenation resulted in superior functional protection when used in combination with the novel mucosal adjuvant, M7. These results provide a new potential strategy for combatting cocaine addiction through mucosal vaccination.

## Introduction

Cocaine is a powerful psychostimulant, the illicit abuse of which has become a serious social and public health concern. It is now estimated that over 14% of Americans aged 12 or older are currently using or have tried cocaine.^1^ Cocaine rapidly enters the brain by crossing the blood brain barrier and exerts its effects upon the central nervous system (CNS). This can occur either through the circulation after injection or through direct transport from the nasal cavity to the brain via the olfactory system when inhaled.^2^ Cocaine blocks the re-uptake of catecholamines, including dopamine, norepinephrine, and serotonin. Roughly 80–90% of cocaine introduced into the body is available to exert its pharmacologic effect, dependent upon the route of administration.^3^ The addictive effects of cocaine are rapid, mediated primarily by its ability to block the dopamine transporter in the CNS, resulting in a profound euphoria that abusers will seek to repeat at cost to their health. Cocaine abuse alters behavior, mood, and can seriously damage the cardiovascular system leading to heart attack and stroke. Death due to cocaine overdose can be sudden and occur before medical intervention is possible. Despite the obvious health risks associated with cocaine abuse, >95% of all cocaine addicts who try to quit fail^4^ and, notwithstanding our wealth of knowledge regarding the basic science of cocaine chemistry and the biology of cocaine addiction, no successful pharmacotherapies have been generated to combat its addictive properties.

The concept of an anti-cocaine vaccine has been proposed as a novel pharmacologic intervention to treat cocaine abuse.^5,6^ In this strategy, a therapeutic cocaine vaccine would be administered to cocaine addicts, resulting in the production of anti-cocaine antibodies that would bind and prevent cocaine from gaining access to the CNS. Key functional read-outs for cocaine vaccine efficacy include the ability of a vaccine to limit the access of cocaine to the nervous system and to block the psychostimulatory effects of cocaine. Although cocaine is a small molecule that would not be capable of generating an antibody response on its own, it has been shown that it can be conjugated to a protein carrier in order to induce anti-cocaine antibodies. Various experimental anti-cocaine vaccines based on this “hapten-carrier” approach have been demonstrated to induce adequate amounts of serum IgG to reduce cocaine levels in the brain, limit cocaine-induced alterations in locomotor activity, and/or reduce cocaine self-administration.^7–10^ In order to generate cocaine-neutralizing antibodies, multiple haptens have been evaluated including cocaine, itself, and the cocaine analog GNC (6-(2R,3S)-3-(benzoyloxy)-8-methyl-8-azabicyclo [3.2.1] octane-2-carbonyloxy-hexanoic acid).^9^ Both of these haptens are capable of generating cocaine-blocking antibodies, but a side-by-side comparison of the immunogenicity of the two hapten candidates has not previously been performed. In humans, phase I and II clinical trials for one anti-cocaine vaccine (TA-CD), which features a cocaine derivative conjugated to the cholera toxin B subunit, have suggested that it was well tolerated.^4,11,12^ Although cocaine-specific antibodies were detected in the sera of immunized individuals for up to 6 months, in general, only 38% of subjects developed high levels of anti-cocaine antibodies.^12^ Those subjects that displayed significantly higher mean antibody titers (those who had received the highest doses of vaccine) were more likely to maintain cocaine-free urine.^4,12^ Collectively, these results suggest that an anti-cocaine vaccine could elicit an immune response that is sufficient to attenuate the effects of cocaine usage in humans. However, current vaccination approaches are not optimal in terms of consistently inducing high titer anti-cocaine responses along all potential routes of cocaine administration (i.e. both in the serum and in mucosal secretions). Neutralizing antibodies to cocaine should ideally be present at the site of cocaine administration in sufficient quantities in order to achieve early and robust neutralization. The difficulty in attaining high titer responses has been identified as a major obstacle for the development of a cocaine vaccine, since higher titer responses were correlated with longer term cocaine abstinence in the TA-CD vaccine study.^4^ One of the principal challenges in the development of anti-cocaine vaccines is to generate effective antibody responses; however, a solution to this problem may lie in the choice of vaccine adjuvant.

Adjuvants are a chemically and structurally heterogeneous group of immuno-stimulatory compounds used to boost immune responses against vaccine antigens. They are necessary in many vaccines since some antigens, such as cocaine, are poorly immunogenic and do not elicit neutralizing immune responses on their own.^13^ A major limitation to progress in the development of anti-cocaine vaccines is the absence of safe and effective adjuvants that can boost and sustain anti-cocaine immune responses in vaccinated individuals. The recent TA-CD cocaine vaccine study employed alum as the adjuvant.^4^ However, this vaccine did not elicit maintained high antibody titers in the serum of all vaccine recipients and, therefore, the vaccine was not consistently protective.^4^ Ideally, a cocaine vaccine would include a mucosal adjuvant that could also protect against an inhaled challenge to the nasal mucosae. Alum is not a mucosal adjuvant and most experimental mucosal adjuvants, such as Cholera toxin, are toxic and not considered candidates for human use.^14^

We have previously reported that small molecule mast cell (MC) activators are extremely effective mucosal adjuvants.^15^ MCs are granulated leukocytes that are present in most vascularized tissues, but especially concentrated in host/environmental interfaces such as the skin or mucosal surfaces.^16^ These cells play an essential role in both the innate and adaptive immune responses during infection. Upon direct or indirect challenge by microbes or microbial components, MCs release their granule contents that contain pre-stored quantities of inflammatory mediators, e.g. TNF, histamine, and tryptase, which are released immediately after activation.^16^ Granule release is followed by the de novo synthesis of large amounts of inflammatory mediators, such as leukotrienes, cytokines, and chemokines, which contribute to the orchestrated mobilization of neutrophils, dendritic cells, and lymphocytes into the challenged tissue site as well as the local draining lymph nodes.^17,18^ Prior studies have shown that co-administration of MC-activating compounds with poorly immunogenic subunit vaccine antigens in the nasal passages of animals (both mice and rabbits) evoked long lasting, high titers of protective antigen-specific IgG in the serum and IgA along mucosal surfaces.^15,19^ Antibodies induced by MC activators, or similar adjuvants designed to replicate MC activity, consistently display high avidity for their antigens, making them extremely effective as neutralizing antibodies.^19,20^ Furthermore, our prior studies have shown that animals immunized with MC activators did not experience any harmful side effects, such as systemic inflammation or localized tissue damage.^15,21^ Some of the most promising MC-activating adjuvants for translational applications are in the mastoparan family. Mastoparan, an insect-derived oligopeptide, and related second-generation analogs, such as M7, are potent activators of MCs.^22–25^

Based on the fact that cocaine is frequently administered via the oral and nasal mucosa and that recent advances have improved in our understanding of mucosal immunity, we set out to develop a cocaine vaccine that would induce robust mucosal responses while also retaining neutralizing ability for a systemic cocaine challenge. The two leading hapten candidates, cocaine and GNC, were each conjugated to the carrier molecule, keyhole limpet hemocyanin (KLH), in order to allow us to assess the anti-hapten responses comparatively. KLH was chosen as the carrier in this system due to its biochemical properties that promote haptenation, as well as existing evidence that it is both immunogenic and safe in humans.^26^ We hypothesized that intranasal co-administration of these carrier protein conjugates with the MC-activating adjuvant compound M7 could be an effective way to evoke high and sustained anti-cocaine antibody levels in both the circulation and mucosal secretions. Here, we also aimed to optimize the route of cocaine vaccine administration and determine whether a nasal route of administration could improve mucosal responses while providing protection from the psychostimulatory effects of cocaine. The results of this study show that nasal immunization against cocaine with a mucosal adjuvant promotes a uniquely effective anti-cocaine response, which emphasizes the role that immunization route and adjuvant can have on imprinting a specialized vaccination response.

## Results

### Design of cocaine vaccine formulations

Considerations for pre-clinical vaccine design for small molecules include the choice of hapten, adjuvant, route of immunization and immunization schedule. For these studies, we compared the activity of two haptens that have the potential to generate cocaine-neutralizing antibodies, cocaine and the cocaine analog GNC,^9^ in eliciting a hapten-specific antibody responses, when conjugated to the carrier molecule, KLH. We also aimed to compare the efficacy of vaccine formulations administered in combination with multiple adjuvants. Since the nasal mucosa represents a major site of cocaine self-administration by users, we tested the ability of the mucosal adjuvant M7 to enhance cocaine-specific antibodies in mucosal secretions, compared to the traditional adjuvant, Alum. Finally, mice were given a cocaine challenge and we measured the ability of each vaccine to limit the increased locomotor activity that follows cocaine exposure. For the complete list of experimental groups and relevant controls, see Table 1. Mice were administered three immunizations in total with an initial priming immunization (Day 0) followed by boosts (Days 14 and 28). Immunizations were given intraperitoneally (i.p.) (for the adjuvant Alum) or nasally (for M7). Additional groups were included where mice were primed with a subcutaneous (s.c.) immunization (containing Alum as the adjuvant) followed by subsequent boosts via the nasal route (with M7 as the adjuvant). This final group was included due to evidence that in some vaccination strategies boosting at an alternate site or via an alternate route could enhance protective responses.^27^

**Table 1:** Cocaine vaccine experimental groups.

Group	Antigen	Prime (Day 0) (adjuvant, route)	Boost (Days 14, 28) (adjuvant, route)
1	None	None	None
2	KLH-cocaine	Alum, i.p.	Alum, i.p.
3	KLH-GNC	Alum, i.p.	Alum, i.p.
4	KLH-cocaine	No adjuvant, nasal	No adjuvant, nasal
5	KLH-GNC	No adjuvant, nasal	No adjuvant, nasal
6	KLH-cocaine	M7-NH2 (20 µg), nasal	M7-NH2 (20 µg), nasal
7	KLH-GNC	M7-NH2 (20 µg), nasal	M7-NH2 (20 µg), nasal
8	KLH-cocaine	Alum, s.c.	M7-NH2 (20 µg), nasal
9	KLH-GNC	Alum, s.c.	M7-NH2 (20 µg), nasal

### Hapten-specific IgG generated during vaccination

**Fig. 1:**
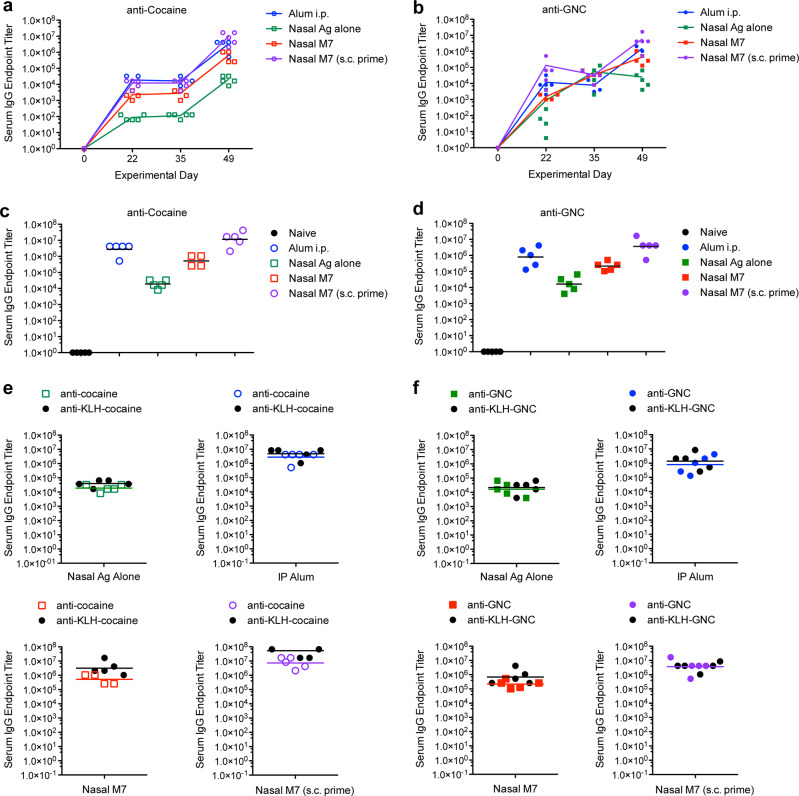
Adjuvants increase hapten-specific IgG titers. **a**, **b** Geometric mean titers for total IgG at designated time points during the immunization protocol for mice immunized with **a** cocaine-KLH or **b** GNC-KLH after administration of vaccines formulated with the designated adjuvants. The geometric mean titers at day 49 of the immunization protocol are presented in panel **c** for cocaine-KLH-immunized groups and in panel **d** for GNC-KLH-immunized groups using a horizontal line. The IgG titers (the horizontal line represents the geometric mean) specific for each hapten were compared to total hapten-conjugate for **e** cocaine-KLH immunized mice and **f** GNC-KLH immunized mice.

Following the experimental immunization protocol, blood was collected and the cocaine-specific or GNC-specific antibodies in serum were measured by ELISA, using cocaine-conjugated BSA or GNC-conjugated BSA, as appropriate, for the capture antigen. All immunized mice seroconverted during the immunization protocol with titers continuing to rise after two vaccination boosts; however, the antigen-specific titers differed significantly by group (Fig. 1a, b). For both haptens, the animals administered hapten-conjugates with Alum, by i.p. injection, or given nasal immunizations with M7 after a s.c. prime containing Alum had the highest hapten-specific serum IgG titers on day 49, at the end of the vaccination protocol (Fig. 1c, d). We observed the same trend when the complete hapten-conjugate (cocaine-KLH or GNC-KLH) was used as the capture antigen for endpoint ELISA measurements (Fig. 1e, f). There was not a statistically significant difference between the titers reached in immunized mouse serum for hapten alone (conjugated to BSA as a carrier to detect specificity) versus the hapten-KLH groups (Fig. 1e, f). This suggests that hapten conjugation to KLH was highly efficient and most of the specific antibodies generated were hapten-specific rather than specific for the KLH carrier protein.

**Fig. 2:**
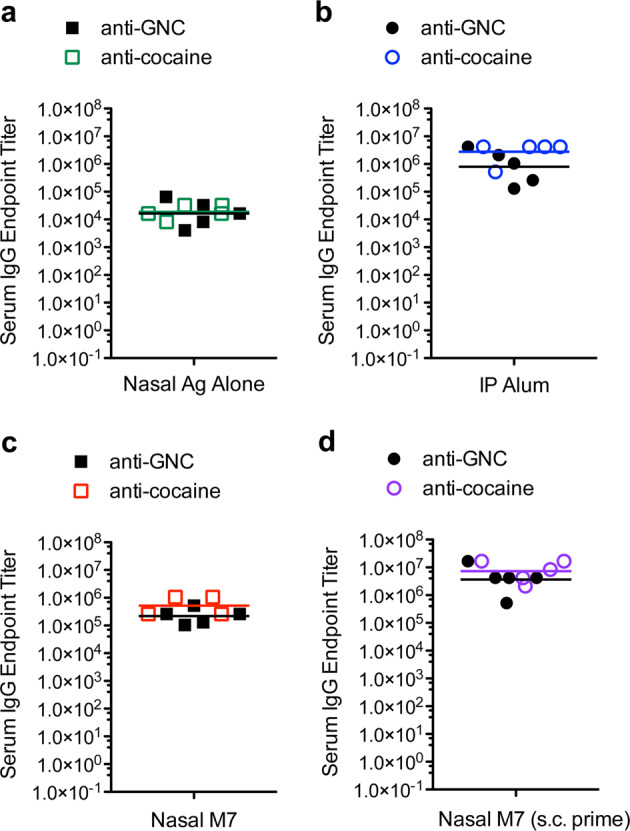
Immunization induced similar IgG titers against the haptens GNC and cocaine. The hapten-specific antibody titers generated against GNC and cocaine did not differ significantly for each adjuvant group **a** antigen alone, administered nasally **b** Alum, administered i.p. **c** M7 administered nasally, and **d** M7 administered nasally after a subcutaneous prime containing Alum. The horizontal line represents the geometric mean.

We also compared the titers of hapten-specific antibodies (anti-GNC versus anti-cocaine) generated for each adjuvant or schedule group. We did not observe a statistically significant difference in the titers of anti-GNC antibodies and anti-cocaine antibodies in analogous groups that included the same adjuvant (Fig. 2). Alum appeared to promote the highest titer antibodies in both of the immunization groups containing this adjuvant (Fig. 1a–d). For each parallel adjuvant and schedule group, hapten conjugates of cocaine and GNC also performed similarly in terms of inducing comparable magnitude specific IgG (Fig. 2).

### Blocking the psychoactive effects of cocaine

**Fig. 3:**
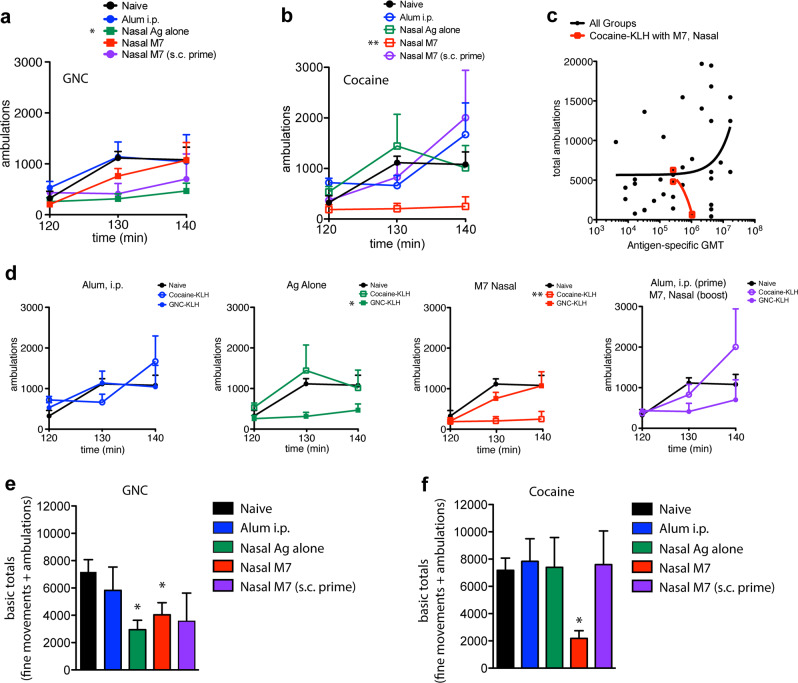
Cocaine-KLH with M7 nasal vaccine most effectively limits cocaine-induced locomotion. Mice were challenged with cocaine (56 mg/kg) over days 56–58. Locomotion after cocaine administration was measured in terms of mean ambulations for each mouse immunization group over time for **a** GNC-KLH immunized and **b** cocaine-KLH immunized mice, compared to naïve mice. Significance was determined by repeated measures ANOVA with Tukey’s Multiple Comparison Test. For both **a**, nasal antigen alone protected significantly (*p* < 0.05); for **b**, nasal M7 protected with highly significant effects (*p* < 0.01). **c** Linear regression was used to determine whether the total ambulations of mice after cocaine challenge were correlated with the antigen-specific GMT. Overall, there was no correlation between antigen-specific titers and ambulations after cocaine challenge. Only for the cocaine-KLH with M7 nasal adjuvant group, there was a strong correlation between high cocaine-specific titers and reduced ambulations with cocaine challenge; Pearson’s *r* = −0.97. **d** Individual comparisons between the average ambulations for cocaine-KLH and GNC-KLH vaccinated groups versus naïve animals after challenge. For cocaine-KLH and GNC-KLH-vaccinated groups, ambulations for individual mice are provided in Figs. S1 and S2, respectively. Total basic movements (the sum of total ambulations and total fine movements) for 30 min after cocaine challenge are displayed for **e** GNC-KLH and **f** Cocaine-KLH groups. * Indicates a significant reduction in basic totals compared to naïve control, cocaine challenged mice by Student’s un-paired *t*-test. Error bars represent the standard error of the mean (SEM).

To determine if any of the cocaine immunization formulations/schedules provided adequate protection from a cocaine challenge, we performed studies to measure mouse locomotor activity after cocaine administration. Animals were habituated in the chambers for 120 min before receiving an i.p. injection of cocaine (56 mg/kg). Thus, 0–120 min was used as a habituation period, and the time after cocaine challenge when naïve mice displayed increased locomotor activity (120–180 min) was considered the period of cocaine challenge. Surprisingly, the results of the behavioral study did not correlate with the antibody titers observed in Fig. 1. Comparing the average total ambulations after cocaine challenge, the cocaine-KLH immunization group administered the vaccine nasally with M7 as the adjuvant provided the strongest and most statistically significant protection in terms of reduced ambulations following cocaine challenge during the 120–180 min challenge period, compared to naïve mice (Fig. 3a, b). Although, overall, there was no correlation between the hapten-specific antibody titers and the total ambulations following cocaine challenge, there was a strong correlation (Pearson’s *r* = 0.97) between high cocaine-specific titers and reduced ambulations for the cocaine-KLH with M7 nasal adjuvant group alone (Fig. 3c). Comparisons of the cocaine vs. GNC hapten vaccination groups are presented in Fig. 3d, and demonstrate that cocaine-KLH antigen with M7 nasal administration had an advantage over GNC-KLH antigen with M7 nasal administration group. The ambulation data for each individual mouse for the duration of the habituation and challenge stages of the experiment are included as Supplementary Fig. 1 for cocaine-KLH immunization groups and as Supplementary Fig. 2 for GNC-KLH immunization groups. A comparison of total ambulations during the challenge period also reflected that the cocaine-KLH vaccine with M7 produced the most robust effect in terms of blocking cocaine-induced locomotion (Supplementary Fig. 3). Since animals that are given high stimulant doses can display stereotypy (which is typified by fine movements rather than ambulations), we also analyzed whether the vaccines were able to prevent total basic movements (the sum of ambulations and fine movements) in the period of time following cocaine challenge (Fig. 3e, f). By this measure, both cocaine-KLH and GNC-KLH vaccines with M7 significantly reduced total basic movements, as did GNC-KLH alone (Fig. 3e, f).

### Cocaine neutralization in the blood and CNS

**Fig. 4:**
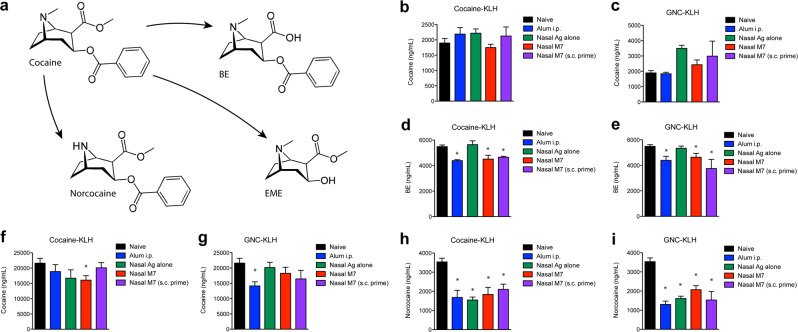
Measurement of cocaine and its metabolic products in the blood and CNS. **a** Structures are provided of cocaine and three of its major metabolic products, BE, EME, and norcocaine, the levels of which were determined in the serum and in the brain by liquid chromatography–electrospray ionization–tandem mass spectrometry. For all panels, **b**–**i** samples were harvested from mice 15 min after a 56 mg/kg challenge with cocaine. Serum cocaine levels were measured in groups of animals vaccinated with **b** Cocaine-KLH (not significant, *p* = 0.38 by ANOVA) and **c** GNC-KLH (not significant, *p* = 0.099 by ANOVA). BE was also measured in the serum of **d** cocaine-KLH and **e** GNC-KLH groups. Cocaine levels in the brain were measured and compared to naïve controls for **f** cocaine-KLH and **g** GNC-KLH groups. For panels **d**–**g**, * indicates a significant difference compared to naïve mice by Student’s unpaired *t* test (*p* < 0.05). Norcocaine levels were significantly reduced in the brains of all **h** cocaine-KLH and **i** GNC-KLH vaccinated groups. For **h** and **i**, * indicates a significant decrease compared to naïve mice (*p* < 0.05). Error bars represent the standard error of the mean (SEM).

To determine if any of the vaccines influenced the concentration of cocaine in the blood, blood was collected 15 min after cocaine administration by i.p. injection and the amounts of cocaine and the cocaine metabolites, benzoylecgonine (BE), ecgonine methyl ester (EME), and Norcocaine were measured by mass spectrometry (Fig. 4a). Cocaine concentration in the blood did not differ significantly between control mice and vaccinated groups (Fig. 4b, c), demonstrating that equivalent amounts of cocaine were delivered to all animals. However, the levels of cocaine metabolite BE were reduced in the blood of all groups of animals that were given adjuvanted vaccines (M7 Nasal group, Alum group and M7 Nasal with s.c. prime) when compared individually to the cocaine levels in the blood of naïve animals (Fig. 4d, e), suggesting that less of the cocaine dose was metabolically active in these vaccinated groups.

A successful anti-cocaine vaccine will prevent cocaine from entering the brain to exert its behavioral effects. Moreover, anti-cocaine antibodies may aid in the clearance of cocaine from the body by sequestering the cocaine in the periphery. Therefore, one of the key measures of the success of an effective anti-cocaine vaccine would be in its ability to prevent cocaine from entering the brain. Reduced cocaine in the brain could explain observed decreases in cocaine-induced ambulations. Again, we examined levels of cocaine and its metabolites in the brain by mass spectroscopy. While BE is the primary cocaine metabolite found in the plasma, norcocaine is the primarily metabolite found in the brain.^28^ When the levels of cocaine in the brains of animals from each vaccinated group were compared directly to the naïve control group, the quantities of cocaine in the brain were significantly reduced in only two groups, the cocaine-KLH with nasal M7 adjuvant group (Fig. 4f) and the GNC-KLH alum group (Fig. 4g). All groups of vaccinated animals, including those vaccinated with hapten-conjugates alone, had reduced brain levels of norcocaine, the primary cocaine metabolite found in the brain (Fig. 4h, i). Collectively, the cocaine-KLH with M7 group and GNC-KLH with alum group performed superior to other vaccine groups since these two vaccines resulted in reduced cocaine levels in the brain and reduced levels of the cocaine metabolites BE in the serum and norcocaine in the brain.

### M7 adjuvant promotes anti-hapten IgA in mucosal secretions

**Fig. 5:**
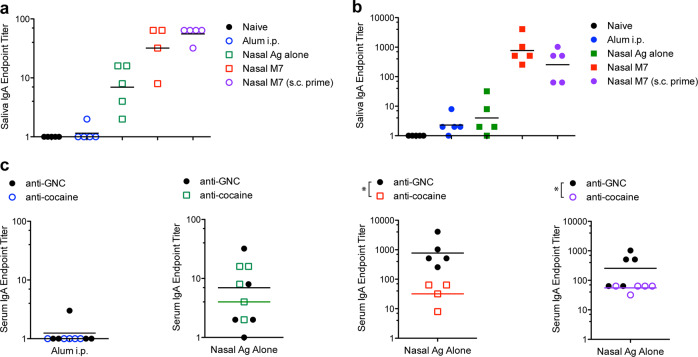
Mucosal adjuvant M7 induces hapten-specific IgA in saliva. **a** Cocaine-specific and **b** GNC-specific IgA were measured in saliva collected day 56 of the experimental protocol. Mucosal administration consistently promoted hapten-specific IgA, with M7 effectively enhancing hapten-specific IgA titers. **c** Comparison of GNC-specific and cocaine-specific IgA titers revealed slightly higher levels of hapten-specific IgA were induced by the GNC hapten-conjugate (*signifies *p* < 0.05 by Student’s un-paired *t* test). The horizontal line represents the geometric mean.

Since cocaine is frequently self-administered directly to the nasal mucosal passages we tested whether the mucosal vaccination strategies could promote anti-cocaine or anti-GNC antibodies in mucosal secretions by measuring anti-hapten IgA in mouse saliva. For both the GNC-KLH and cocaine-KLH antigens, vaccination schedules that included M7 as an adjuvant induced high titer hapten-specific IgA (Fig. 5a, b). This also occurred independent of whether the priming vaccination was intra-nasal or sub-cutaneous (Fig. 5a, b). The alum-adjuvanted vaccines failed to induce anti-hapten IgA; indeed, the average specific IgA titers in these groups were even lower than in groups vaccinated with unadjuvanted hapten-conjugates alone (Fig. 5a, b). When compared side-by-side, IgA titers were also moderately higher towards the hapten GNC than towards cocaine (Fig. 5). Thus, nasal administration of cocaine-KLH or GNC-KLH with M7 was highly effective in inducing IgA titers in all groups where this route of administration and adjuvant were included in the immunization protocol.

## Discussion

In this study, we have developed a novel vaccination approach that induces strong humoral and mucosal immunity to cocaine. This vaccine is able to reduce cocaine translocation into the brain and block its psychoactive effects. Using a combinatorial approach to compare the efficacy of vaccines containing either the hapten cocaine or GNC and multiple adjuvants we have identified key attributes of these cocaine vaccine formulations that confer stronger protection to cocaine-challenged animals. Our results show that cocaine-KLH antigen with M7 as a nasal adjuvant was the strongest performing vaccine from our panel in terms of limiting behavioral changes after cocaine injection. All M7-adjuvanted vaccines also induced higher avidity antibodies against cocaine than the standard adjuvant, Alum, and promoted the production of hapten-specific IgA (either against cocaine or GNC) in mucosal secretions. The potency of the nasal adjuvant activity of M7 agrees with recent results from our laboratory where M7 was used as a nasal vaccine adjuvant for HIV-1 gp120 in mice, rabbits and non-human primates.^29^ Due to the common practice of administering cocaine to mucosal surfaces, the ability to promote strong, specific IgA responses against cocaine is a key advantage of a mucosal vaccine strategy and has the potential to more effectively neutralize cocaine that is administered via the nasal or oral mucosae.^4^ Future studies may be required to evaluate the degree to which IgA can contribute to protection against a mucosal cocaine challenge. Interestingly, IgA is also present in cerebrospinal fluid;^30^ thus it is possible that IgA inside the blood brain barrier could have contributed to cocaine neutralization in our study. In general, vaccination strategies designed to boost IgA production, such as this one, may have advantages for neutralization of psychostimulants due to the ability to generate antibodies inside the CNS.

Interestingly, the magnitude of the serum-IgG titer following vaccination did not accurately reflect the degree of protection from cocaine challenge afforded by the vaccination schedule. Other studies have presumed that the magnitude of the specific antibody titer was the key determinant for cocaine vaccination effectiveness, since patients that had higher cocaine-specific antibody titers also apparently had reduced cocaine usage.^4,11^ High titer antibodies are thought to be required to ensure neutralization of cocaine before it is able to cross the blood–brain barrier.^4^ However, our data suggests that other aspects of the humoral response aside from the magnitude of antibody responses strongly influence cocaine neutralization in vivo, especially with regards to abrogating cocaine-induced behavioral changes. Potentially, these other aspects could relate to the antibody-binding characteristics, the breadth of the antibody response, or the subclasses of antibodies that are induced. These factors can each be influenced differently by unique adjuvants.^31^ Particulate adjuvants that target lymph nodes and are designed to simulate the granules that MCs exocytose during activation have been shown to improve the antibody avidity compared to traditional adjuvants such as alum, for example.^20^ Here, alum was effective in inducing high titer antibodies but these antibodies were less effective functionally in vivo in blocking cocaine-induced ambulations. Even including a prime with Alum prior to a boost with M7 reduced the vaccine effectiveness compared to nasal vaccination including M7 alone, in spite of achieving an extremely high titer antibody response to cocaine. A similar effect where serum IgG titer did not correlate with neutralization was observed in a previous study with nasal immunization for anthrax recombinant protective antigen in conjunction with various adjuvants.^32^ Notably, compound 48/80, another MC-activating compound, was the most effective adjuvant for inducing toxin neutralization in that study.^32^ The results of these multiple studies emphasize the potential of MC activation to enhance antibody quality (i.e. avidity or neutralization) during vaccination. Our results appear to contradict the existing assumption that the primary aim of cocaine vaccination strategies should be to generate the highest titer antibodies possible. Since antibody titers inevitably wane in the months and years following vaccination, these findings provide information that there is scope for achieving protection with high quality antibodies, even at sub-maximal titer.

In spite of generating similar titer IgG antibodies against their respective haptens, the cocaine-KLH antigen outperformed GNC-KLH in both its ability to reduce locomotor activity after cocaine challenge and to limit cocaine passage into the brain. Thus, it is likely that GNC-KLH antibodies have reduced neutralizing capacity against cocaine than antibodies generated against cocaine-KLH. Other studies have shown that GNC can also effectively induce antibodies that can limit the psychostimulatory ability of cocaine,^8^ but we are not aware of a published side-by-side comparison of these two hapten-carrier strategies. Other strategies have also been employed recently and were shown to be effective in reducing cocaine-induced locomotion, using a strategy of haptenating metabolites GNE or GND to tetanus toxoid but those were not compared here.^33^ Here, for the mice in the GNC-KLH group there also appeared to be individual mice that were highly protected while others were non-responders. It is possible that giving additional boosts of the GNC-KLH with M7 vaccine (or other GNC-based groups) could also eventually generate effective neutralizing responses. However, importantly, the protection of the cocaine-KLH with M7 vaccine was more uniform for the individuals in this group and apparent after only two vaccine boosts. Reducing the duration of the vaccination protocol and the delay before the vaccine is effective is a particularly strong consideration for the target population of individuals with psychostimulant addiction. Thus, we anticipate that cocaine-KLH is a more effective vaccine antigen for rapid acquisition of cocaine-neutralizing antibodies in vivo.

Further studies may be needed to determine why some mice responded effectively in terms of functional protection to the GNC-KLH vaccine while others did not. The presence of some responders and non-responders in the group that was immunized with GNC-KLH nasally with M7 may also explain why cocaine was significantly reduced in the brains of mice, yet there were not statistically significant reductions in cocaine-induced locomotor activity, over all. The other vaccine group that reduced cocaine levels in the brains of mice was immunized with Cocaine-KLH with M7, suggesting that it promotes sequestration of cocaine outside the CNS. This vaccine also reduced levels of the metabolic product norcocaine in the brain. In contrast, cocaine levels did not differ significantly in the serum of challenged animals. The observation that less of the cocaine metabolic product BE was generated in the serum of mice (of multiple vaccinated groups, including the optimally performing cocaine-KLH with M7 group) suggests that the bioavailability of cocaine in the serum may be reduced by vaccination. These findings are also consistent with sequestering of antibody bound cocaine in the blood without access to the CNS. One caveat to the use of cocaine metabolites to assess vaccine protection is that specific antibodies to cocaine might also cross-react with any of the metabolic products, given the similarities between structures of cocaine and its metabolites. Thus, functional protection against cocaine-induced locomotion is likely to be the clearest read-out of cocaine inhibition.

In summary, the optimal cocaine vaccine formulation identified in our study was cocaine-KLH as antigen in combination with the novel mucosal adjuvant M7, given via the nasal immunization route with a nasal rather than s.c. prime, due to its combined ability to limit cocaine-induced ambulations, reduce detection of cocaine in the brain, and to generate both cocaine-specific IgG and IgA responses. The key strength of the adjuvant over other strategies appeared to be its capacity to generate high avidity cocaine-neutralizing antibodies. This, surprisingly, provided more robust protection from cocaine challenge than vaccines that induced the highest titer antibodies. M7 is a promising developmental-stage mucosal adjuvant that offers key advantages in terms of generating mucosal immunity while simultaneously promoting high quality neutralizing antibodies.

## Methods

### Animals and vaccinations

Male BALB/c mice were purchased from The Jackson Laboratory (Bar Harbor, ME) at 4–6 weeks of age. Mice were housed in ventilated plastic cages with ad libitum food and water on a 12-h light/dark cycle (lights on at 6:00 a.m.). Mice were vaccinated by intranasal or s.c. or i.p. immunization. Vaccine formulations were administered on days 0, 14, and 28. Either KLH-cocaine or KLH-GNC^9^ (72 μg) was utilized as antigen for all vaccinations. Before nasal vaccination, mice were anesthetized with isoflurane (Butler Schein, Dublin, OH). Vaccine formulations were administered intranasally in 10.5 μl per nostril with M7-NH2 (20 μg) as adjuvant. For s.c. or i.p. immunizations with Alum as the adjuvant, Alum mixed at a 1:1 ratio with antigen.

### Sample collection

Unanesthetized mice were bled from the submandibular vein and the blood samples were collected into 1.5 ml centrifuge tubes. After centrifuging at 16,060 × *g* at 4 °C for 20 min, the serum supernatant was pipetted off and frozen at −80 °C for subsequent analysis. To induce salivation, 3 µg of carbamycholine chloride (Sigma) was administered i.p. and saliva was collected. After centrifuging the collected saliva at 16,060 × *g*, supernatant was transferred into fresh tube and similarly frozen at −80 °C for subsequent analysis.

### Serology

ELISAs were performed as described in previously.^20,34^ In brief, to measure cocaine and GNC-specific IgG and IgA antibodies in serum and saliva, a standard ELISA using alkaline phosphatase-conjugated anti-mouse IgG (Cat. #1030-04;Southern Biotech, Birmingham, AL) and anti-mouse IgA (Cat. 1040-04, Southern Biotech, Birmingham, AL) antibodies were used at the manufacturer’s recommended dilution. BSA-cocaine (3 µg) or BSA-GNC (3 µg), diluted in carbonate/bicarbonate buffer, were added to 96-well ELISA plates as coating antigen. Alkaline phosphatase-anti-mouse IgG or IgA was for detection antibody, and AttoPhos substrate (Promega, Madison, WI) was for developing reagent. Samples were diluted 1:4 or 1:64 (saliva or serum, respectively) followed by additional two-fold dilutions to determine the end-point titer. Sample end-point titers were calculated as the last dilution at which the sample relative light unit reading (560 nm) was three-fold greater than a similarly diluted naïve sample relative light unit reading (560 nm). The ELISA titers presented in the manuscript represent geometric mean titers with log_2_ titers used for statistical analysis.

### Drugs and treatments

Cocaine HCl was provided by the Research Triangle Institute (Research Triangle Park, NC), courtesy of the National Institute of Drug Abuse. Cocaine was dissolved in saline and administered by i.p. injection. Following habituation, 56 mg/kg of cocaine was given to control or immunized mice.

### Behavior

Mice were placed in 40 × 40 × 40 cm photobeam devices (Kinder Scientific, Poway, CA) to measure motor activity. Mice were allowed to explore this open-field for 2 h prior to injection of 56 mg/kg cocaine. Cocaine effects on activity were recorded for 1 h after challenge. Software supplied by the manufacturer summarized photocell interruptions as ambulations or fine movements.^35^ Data were summed in 10 min intervals and group averages are shown. The experimenter was blind to vaccine treatments.

### Tissue collection

One week after the locomotor behavior determination, mice were again dosed with 56 mg/kg cocaine as before. After 15 min, mice were anesthetized with isoflurane and blood was collected via cardiac puncture, placed into tubes containing 10 µl NaF and centrifuged at 4 °C. Mice were decapitated and whole brain was removed and rapidly frozen on dry ice. Brains and sera were stored at −80 °C. Samples were sent for analysis of cocaine, BE, EME, and norcocaine by liquid chromatography–electrospray ionization–tandem mass spectrometry using published protocols,^36,37^ courtesy of Drs. David Moody, David Andrenyak, and Wenfang Fang at the University of Utah.

### Statistics

One-way ANOVA with either Bonferroni’s post-test to compare all groups or Dunnet’s post-test to compare all groups to the control was used. For endpoint titers which are not normally distributed, the data was transformed to log values before statistical analysis. For all experiments *n* = 5 unless otherwise noted. All data are presented as the means of experimental replicates using individual mice and error bars represent the SEM throughout the manuscript. Data were organized for analysis using Excel software and plotted and statistical tests were performed in Prism software.

### Ethical considerations

All animal procedures complied with relevant ethical regulations and were approved by the Duke University Institutional Animal Care and Use Committee (IACUC).

### Reporting summary

Further information on research design is available in the Nature Research Reporting Summary linked to this article.

## Supplementary information

Supplementary Information

Reporting Summary

## Data Availability

The datasets generated during and/or analyzed during the current study are available from the corresponding author on reasonable request.

## References

[1] Gordon S, Clarke S, Greaves D, Doyle A (1995). Molecular immunobiology of macrophages: recent progress. [Review]. Curr. Opin. Immunol..

[2] Chow HS, Chen Z, Matsuura GT (1999). Direct transport of cocaine from the nasal cavity to the brain following intranasal cocaine administration in rats. J. Pharm. Sci..

[3] Goldstein RA, DesLauriers C, Burda AM (2009). Cocaine: history, social implications, and toxicity—a review. Dis. Mon..

[4] Orson, F. M., Kinsey, B. M., Singh, R. A., Wu, Y. & Kosten, T. R. Vaccines for cocaine abuse. *Hum. Vaccin.* **5**. 10.4161/hv.5.4.7457 (2009). 10.4161/hv.5.4.7457 PMC2878138 19276665

[5] Bagasra O, Forman LJ, Howeedy A, Whittle P (1992). A potential vaccine for cocaine abuse prophylaxis. Immunopharmacology.

[6] Fox BS (1996). Efficacy of a therapeutic cocaine vaccine in rodent models. Nat. Med..

[7] Carrera MR, Ashley JA, Wirsching P, Koob GF, Janda KD (2001). A second-generation vaccine protects against the psychoactive effects of cocaine. Proc. Natl Acad. Sci. USA.

[8] Carrera MR (2000). Cocaine vaccines: antibody protection against relapse in a rat model. Proc. Natl Acad. Sci. USA.

[9] Carrera MR (1995). Suppression of psychoactive effects of cocaine by active immunization. Nature.

[10] Koetzner L (2001). Titer-dependent antagonism of cocaine following active immunization in rhesus monkeys. J. Pharmacol. Exp. Ther..

[11] Kosten TR (2002). Human therapeutic cocaine vaccine: safety and immunogenicity. Vaccine.

[12] Martell BA (2009). Cocaine vaccine for the treatment of cocaine dependence in methadone-maintained patients: a randomized, double-blind, placebo-controlled efficacy trial. Arch. Gen. Psychiatry.

[13] Mitchison NA (1971). The carrier effect in the secondary response to hapten-protein conjugates. II. *Cellular cooperation*. Eur. J. Immunol..

[14] Pavot V, Rochereau N, Genin C, Verrier B, Paul S (2012). New insights in mucosal vaccine development. Vaccine.

[15] McLachlan JB (2008). Mast cell activators: a new class of highly effective vaccine adjuvants. Nat. Med..

[16] Galli SJ, Nakae S, Tsai M (2005). Mast cells in the development of adaptive immune responses. Nat. Immunol..

[17] Abraham SN, St John AL (2010). Mast cell-orchestrated immunity to pathogens. Nat. Rev. Immunol..

[18] Kunder CA (2009). Mast cell-derived particles deliver peripheral signals to remote lymph nodes. J. Exp. Med..

[19] Staats HF (2011). Mucosal targeting of a BoNT/A subunit vaccine adjuvanted with a mast cell activator enhances induction of BoNT/A neutralizing antibodies in rabbits. PLoS ONE.

[20] St John AL, Chan CY, Staats HF, Leong KW, Abraham SN (2012). Synthetic mast-cell granules as adjuvants to promote and polarize immunity in lymph nodes. Nat. Mater..

[21] McGowen AL, Hale LP, Shelburne CP, Abraham SN, Staats HF (2009). The mast cell activator compound 48/80 is safe and effective when used as an adjuvant for intradermal immunization with Bacillus anthracis protective antigen. Vaccine.

[22] King TP, Jim SY, Wittkowski KM (2003). Inflammatory role of two venom components of yellow jackets (*Vespula vulgaris*): a mast cell degranulating peptide mastoparan and phospholipase A1. Int. Arch. Allergy Immunol..

[23] Mukai H, Suzuki Y, Kiso Y, Munekata E (2008). Elucidation of structural requirements of mastoparan for mast cell activation-toward the comprehensive prediction of cryptides acting on mast cells. Protein Pept. Lett..

[24] Johnson, B. T., Kulis, M., Abraham, S. N., Burks, A. W. & Staats, H. F. Nasal immunization with peanut antigen and the cationic peptide adjuvant mastoparan 7 induces serum humoral immunity that protects peanut allergic mice against systemic anaphylaxis. *J. Allergy Clin. Immunol.* **129**, AB176. 10.1016/j.jaci.2011.12.221 (2012).

[25] Shelburne C, Li G, Staats H, Abraham S (2010). Development of a novel anti-FimH vaccine using a mast cell activator as the adjuvant. J. Immunol..

[26] Milgrom H (2012). Response to cutaneous immunization with low-molecular-weight subunit keyhole limpet hemocyanin. Int. Arch. Allergy Immunol..

[27] Fiorino F, Pettini E, Pozzi G, Medaglini D, Ciabattini A (2013). Prime-boost strategies in mucosal immunization affect local IgA production and the type of th response. Front. Immunol..

[28] Bystrowska B (2012). LC/MS/MS evaluation of cocaine and its metabolites in different brain areas, peripheral organs and plasma in cocaine self-administering rats. Pharmacol. Rep..

[29] Jones, D. I. et al. Optimized mucosal modified vaccinia virus Ankara prime/soluble gp120 boost HIV vaccination regimen induces antibody responses similar to those of an intramuscular regimen. *J. Virol*. **93**. 10.1128/jvi.00475-19 (2019). 10.1128/JVI.00475-19 PMC6600201 31068425

[30] Sindic CJ, Delacroix DL, Vaerman JP, Laterre EC, Masson PL (1984). Study of IgA in the cerebrospinal fluid of neurological patients with special reference to size, subclass and local production. J. Neuroimmunol..

[31] Kenney JS, Hughes BW, Masada MP, Allison AC (1989). Influence of adjuvants on the quantity, affinity, isotype and epitope specificity of murine antibodies. J. Immunol. Methods.

[32] Gwinn WM (2013). A comparison of non-toxin vaccine adjuvants for their ability to enhance the immunogenicity of nasally-administered anthrax recombinant protective antigen. Vaccine.

[33] Kimishima A, Olson ME, Natori Y, Janda KD (2018). Efficient syntheses of cocaine vaccines and their in vivo evaluation. ACS Med. Chem. Lett..

[34] Nordone SK, Peacock JW, Kirwan SM, Staats HF (2006). Capric acid and hydroxypropylmethylcellulose increase the immunogenicity of nasally administered peptide vaccines. AIDS Res. Hum. Retroviruses.

[35] Caster JM, Walker QD, Kuhn CM (2007). A single high dose of cocaine induces differential sensitization to specific behaviors across adolescence. Psychopharmacology.

[36] Walsh SL, Stoops WW, Moody DE, Lin SN, Bigelow GE (2009). Repeated dosing with oral cocaine in humans: assessment of direct effects, withdrawal, and pharmacokinetics. Exp. Clin. Psychopharmacol..

[37] Lin SN, Moody DE, Bigelow GE, Foltz RL (2001). A validated liquid chromatography-atmospheric pressure chemical ionization-tandem mass spectrometry method for quantitation of cocaine and benzoylecgonine in human plasma. J. Anal. Toxicol..

