# Supplementary material for: Novel mucosal adjuvant, mastoparan-7, improves cocaine vaccine efficacy
Source: NPJ Vaccines. 2020 Feb 5;5:12. doi: 10.1038/s41541-020-0161-1 (PMC7002721; doi:10.1038/s41541-020-0161-1)
Supplement: Supplementary file 1 — Supplementary Information [file 41541_2020_161_MOESM1_ESM.pdf]

Supplementary Information

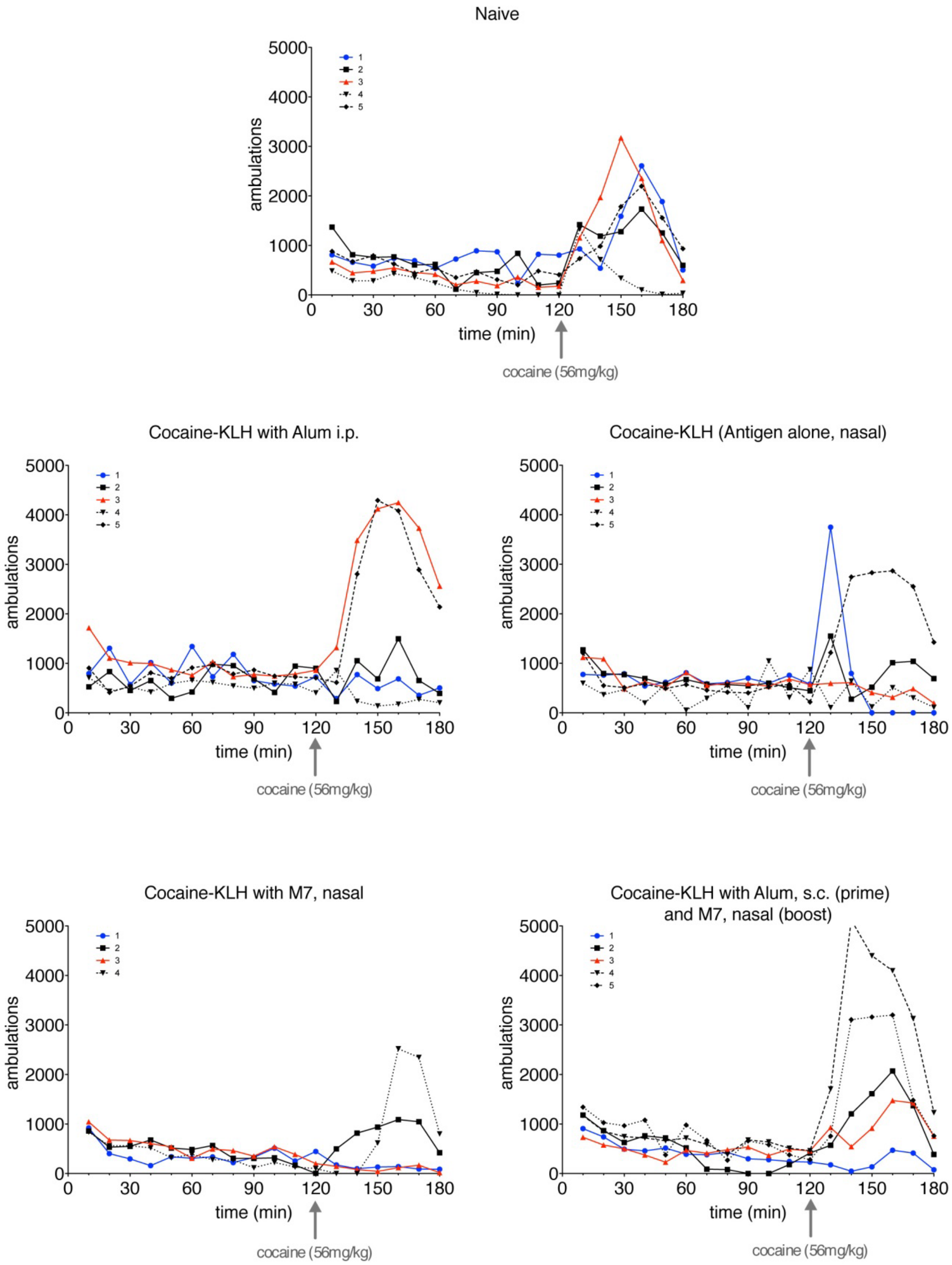

Supplementary Figure 1: Cocaine-induced ambulations for individual Cocaine-KLH vaccinated mice. Ambulations were recorded during the habituation period and after cocaine challenge.

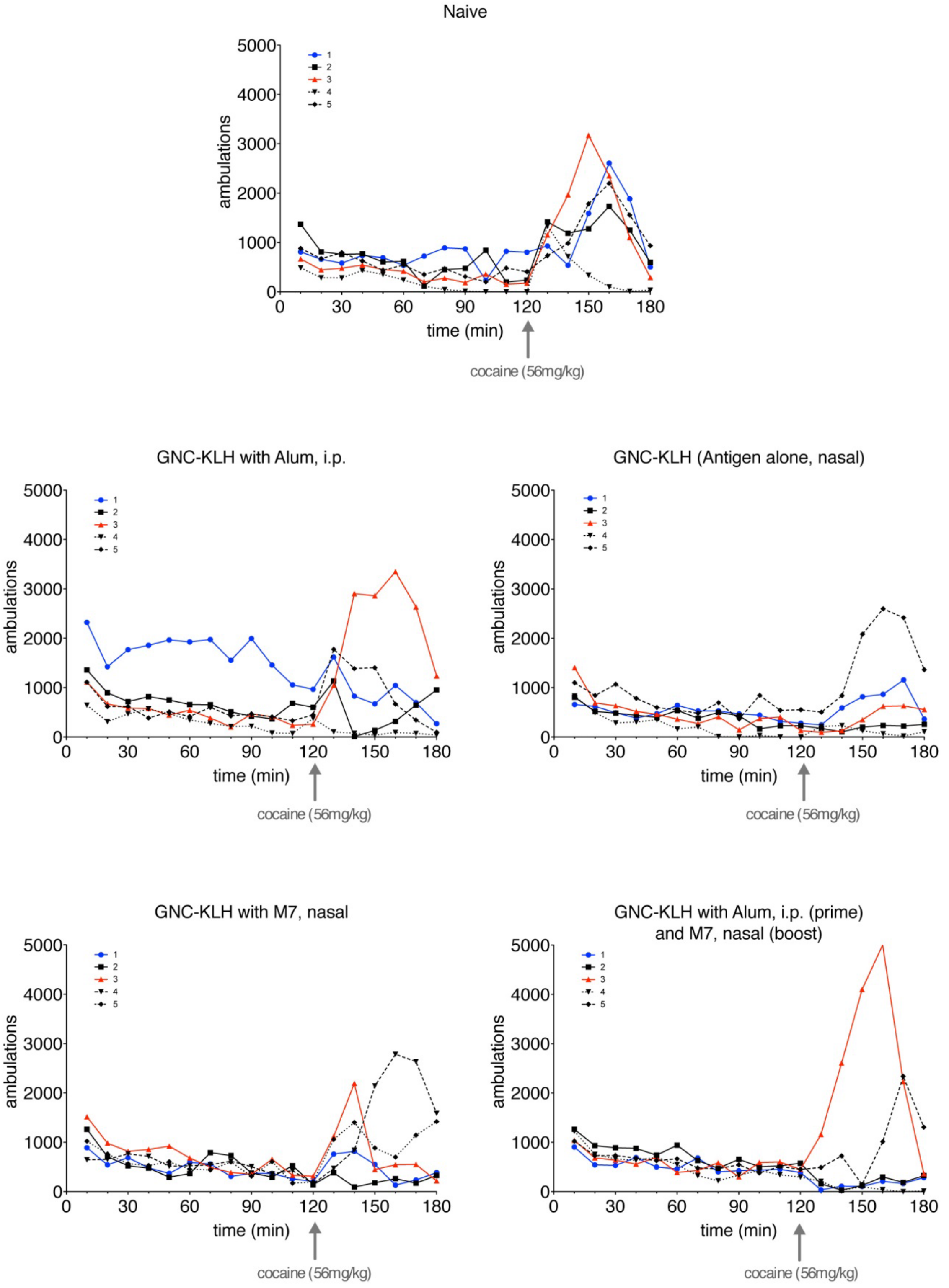

Supplementary Figure 2: Cocaine-induced ambulations for individual GNC-KLH vaccinated mice.
Ambulations were recorded during the habituation period and after cocaine challenge.

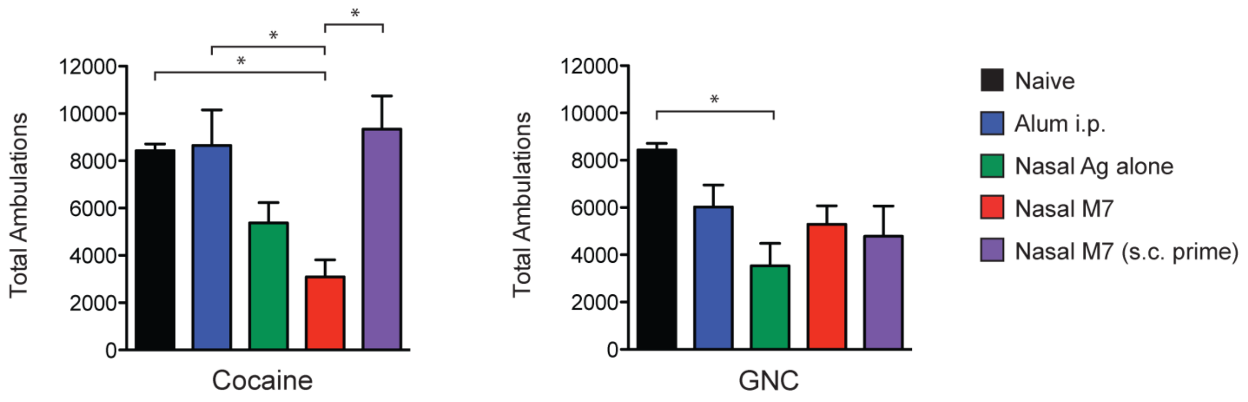

Supplementary Figure 3. Cocaine-KLH vaccine with M7 robustly blocked cocaine-induced ambulations. The total ambulations recorded over the challenge period (120 to 180 minutes) are presented on the graphs for the antigens cocaine-KLH and GNC-KLH with the designated antigens. Significance was determined by 1-way ANOVA with Tukey's multiple comparison test to determine significance between groups.
